# Folding of VemP into translation-arresting secondary structure is driven by the ribosome exit tunnel

**DOI:** 10.1093/nar/gkac038

**Published:** 2022-02-12

**Authors:** Michal H Kolář, Gabor Nagy, John Kunkel, Sara M Vaiana, Lars V Bock, Helmut Grubmüller

**Affiliations:** Department of Theoretical and Computational Biophysics, Max Planck Institute for Multidisciplinary Sciences, Am Fassberg 11, 370 77 Göttingen, Germany; Department of Physical Chemistry, University of Chemistry and Technology in Prague, Technická 5, 166 28 Prague, Czech Republic; Department of Theoretical and Computational Biophysics, Max Planck Institute for Multidisciplinary Sciences, Am Fassberg 11, 370 77 Göttingen, Germany; Department of Physics and Center for Biological Physics, Arizona State University, Tempe, AZ 85287, USA; Department of Physics and Center for Biological Physics, Arizona State University, Tempe, AZ 85287, USA; Department of Theoretical and Computational Biophysics, Max Planck Institute for Multidisciplinary Sciences, Am Fassberg 11, 370 77 Göttingen, Germany; Department of Theoretical and Computational Biophysics, Max Planck Institute for Multidisciplinary Sciences, Am Fassberg 11, 370 77 Göttingen, Germany

## Abstract

The ribosome is a fundamental biomolecular complex that synthesizes proteins in cells. Nascent proteins emerge from the ribosome through a tunnel, where they may interact with the tunnel walls or small molecules such as antibiotics. These interactions can cause translational arrest with notable physiological consequences. Here, we studied the arrest caused by the regulatory peptide VemP, which is known to form α-helices inside the ribosome tunnel near the peptidyl transferase center under specific conditions. We used all-atom molecular dynamics simulations of the entire ribosome and circular dichroism spectroscopy to study the driving forces of helix formation and how VemP causes the translational arrest. To that aim, we compared VemP dynamics in the ribosome tunnel with its dynamics in solution. We show that the VemP peptide has a low helical propensity in water and that the propensity is higher in mixtures of water and trifluorethanol. We propose that helix formation within the ribosome is driven by the interactions of VemP with the tunnel and that a part of VemP acts as an anchor. This anchor might slow down VemP progression through the tunnel enabling α-helix formation, which causes the elongation arrest.

## INTRODUCTION

In all organisms, proteins are synthesized by ribosomes. Each ribosome consists of several strands of ribonucleic acid (RNA) and proteins organized in two ribosomal subunits. Ribosomes translate the genetic information stored in messenger RNA into a sequence of amino acids (AAs). The small subunit reads the information, the large subunit catalyzes peptide bond formation. One by one, AAs are delivered to the ribosome by transfer RNAs (tRNAs) and are covalently bound to the nascent peptide chain (NC) at the peptidyl-transferase center (PTC) of the ribosome.

The PTC is buried deep within the large subunit and each NC leaves the ribosome through a 10 nm long exit tunnel. Recent studies have suggested an active role of the tunnel in protein synthesis and translation regulation (1,2). Some NCs interact with the tunnel walls such that peptide-bond formation is slowed down or completely inhibited.

The elongation arrest can have many physiological consequences. For instance, some stalling NCs were shown to stall on faulty mRNAs (3,4) or to serve as chemical (5–8) or mechanical (9,10) sensors. Electrostatic interactions between the NC and the negatively charged exit tunnel were shown to reduce the rate of peptide elongation (11). In particular, positively charged AAs cause transient elongation arrest (11). In addition, macrolides, a large class of antibiotics, act by binding inside the ribosome tunnel and cause stalling (12).

The inherent flexibility of NCs complicates their structure determination by classic biophysical techniques such as X-ray crystallography or cryogenic electron microscopy (cryo-EM). Nevertheless, several ribosome·nascent-chain (RNC) complexes have been resolved, taking advantage of the elongation arrest (5–7,13–18). Computer simulations can be used to complement structural data by dynamics and energetics and to probe the unstalled conformations (16,18–21).

A recently discovered stalling NC is the Vibrio export-monitoring peptide (VemP) (10). In the marine-estuarine bacterium *Vibrio alginolyticus*, VemP regulates the expression of a gene variant via an elongation arrest, thus enabling the bacterium to survive in fresh as well as in sea water (10). The structure of the nascent VemP peptide in the tunnel is controlled by an external mechanical force acting on the N-terminus of the peptide. When the force, presumably generated by the translocon channel, is acting on the VemP peptide at the tunnel exit, the peptide is most likely extended in the ribosome tunnel and translation proceeds unperturbed (21,22). In the absence of this force, however, VemP adopts a compact structure, as recently discovered by cryo-EM (17).

**Figure 1.**
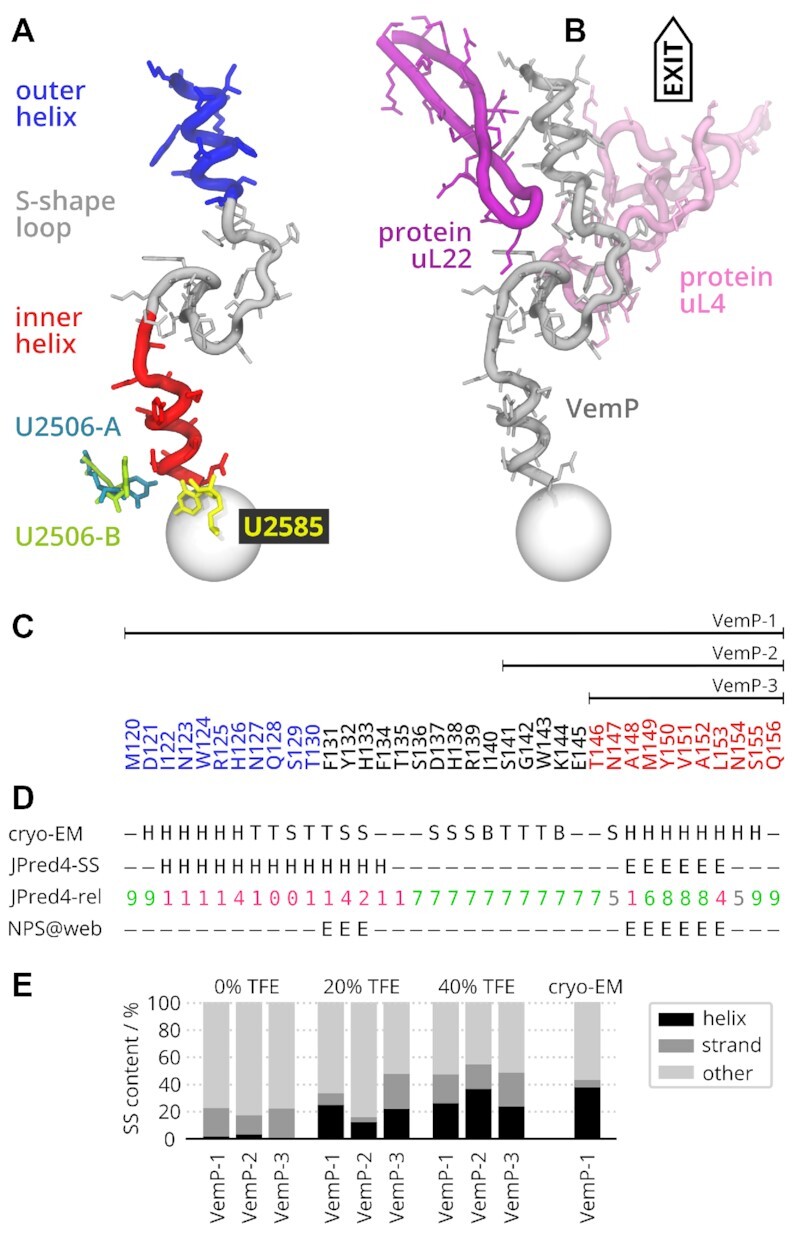
Overview and secondary structure (SS) characteristics of VemP constructs. (**A**) Relevant VemP parts with the critical nucleotides at the peptidyl trasferase center (sphere). Two substates of U2506 are denoted -A and -B. (**B**) VemP stalling conformation (17) in the context of two exit-tunnel proteins uL4 and uL22. (**C**) Primary structure and a schematic representation of the studied constructs. The VemP sequence is color coded (blue = outer helix, red = inner helix). (**D**) Structure-based classification by DSSP (33) for the cryo-EM model (H = α-helix, T = turn, B = β-bridge, S = bend) and sequence-based SS predictions of VemP-1 derived by JPred4 (34,35) (H = helix, E = strand) with the reliability values (JPred4-rel, larger values in green are more reliable than low values in red) and NPS@web (36) (E = strand). (**E**) Secondary structure (SS) content of three VemP constructs as inferred by SESCA analysis of CD spectra in three solvents differing in 2,2,2-trifluorethanol (TFE) molar fraction. For comparison, the SS content of the VemP cryo-EM model (17) in the ribosome tunnel is shown as classified by DSSP.

The atomic model of the ribosome-VemP complex, derived from cryo-EM densities determined at an average resolution of 2.9 Å, revealed that VemP folds into two α-helices connected via an S-shaped loop (Figure 1A). The inner helix is located at the PTC, whereas the outer helix occupies a wider corridor located beyond the constriction of the tunnel formed by extensions of ribosomal proteins uL4 and uL22 (Figure 1B).

Based on the cryo-EM data, it was proposed that the inner helix induces a conformational change of the PTC nucleotides U2506 and U2585 (*Escherichia coli* numbering). This conformation is sterically incompatible with the accommodation of tRNA into the A site and thus prevents the delivery of a new AA (17) (phenylalanine in the VemP case). Moreover, in the cryo-EM model, two substates of U2506 were observed, denoted *A* and *B*, each with an occupancy of 50%. Nucleotide U2506 in substate A occupies the volume of the incoming phenylalanine, and in substate B, U2506 shifted the the N-terminus of the inner helix. The role of the two substates is not fully understood, however.

VemP contains 159 AAs and stalls when the codon of Q156 resides in the P site. In two papers, Ito *et al.* performed single-point mutation experiments with VemP. Using radiolabeled toeprinting *in vitro* (10) and LacZ assays *in vivo* (23), they showed that VemP is remarkably sensitive to mutations. By measuring the activity of the LacZ reporter of VemP mutants, they found a pattern with a periodicity of three or four AAs at the C-terminus (23) that correlates with the α-helical structure of the stalling motif as determined by cryo-EM. The stalling motif, defined by about 20 AAs (S136–Q156), is longer by more than 10 AAs than other stalling peptides like SecM (14,24), TnaC (5,6), ErmBL (16) or MifM (9).

Here, we ask which molecular driving forces cause VemP α-helix formation in the ribosome tunnel. Previously it was argued that the tunnel features ‘folding zones’, where a compaction of the NC occurs. The region nearest to the PTC was identified as having the strongest compaction effect on model polyalanines (25). Helices of a voltage-gated K^+^ channel were shown to fold only in the vestibule near the tunnel exit (26). By *in vitro* translation experiments and molecular dynamics (MD) simulations it was also shown that hydrophobic helices are more likely to fold in the ribosome tunnel than hydrophilic helices (27). The compaction of NCs is, however, not limited to helical conformations, as suggested by experimental assays of NCs with introduced Trp, Ala or Phe. Other compact motifs like loops or kinks may occur in the ribosome tunnel too (28).

A coarse-grained off-lattice model in a non-specific confined space suggested that helix formation is favorable, and entropically driven (29). This, however, contradicts MD simulations performed by Sorin and Pande (30), who argued that helices are destabilized by a cylindrical (carbon nanotube-like) space. The dominant role in the confinement was attributed to the entropy of water (30).

Water represents the major content of the ribosomal exit tunnel. Its walls—mainly composed of rRNA augmented by AAs of ribosomal proteins—confine water close to them. Computational studies of the exit tunnel indicated a complex solvent environment that differs from bulk water in a position-dependent manner (31,32). Lucent *et al.* found that the water in the exit tunnel is a slow-moving (viscous) medium with relative permittivity ϵ_*r*_ between that of bulk water and structured water of macromolecules. In addition, the authors showed that the part of the tunnel located between the PTC and the constriction site, where the helical structure of VemP is formed, has the lowest water mobility (20–30% of the bulk), but a permittivity ϵ_*r*_ close to bulk water favoring polar contacts.

Here, we investigate the molecular details of the VemP ribosomal stalling mechanism. To determine what drives VemP secondary structure (SS) formation within the exit tunnel, we determined and compared the inherent SS propensities of VemP in the ribosome as well as outside, in solution. We estimated VemP SS propensities based on its sequence and performed all-atom MD simulations of several constructs of wild type VemP. Further, we complemented the computational results with circular dichroism (CD) spectroscopy measurements in various solvents. Finally, we investigated the role of both U2506 substates in the elongation arrest by running separate sets of MD simulations.

## MATERIALS AND METHODS

### Studied molecular systems

We studied three VemP constructs whose sequences are denoted by VemP-1, VemP-2 and VemP-3 (Figure 1C). VemP-1 covers residues M120–Q156, which have been resolved in the ribosome tunnel by cryo-EM (17), and comprise inner helix, S-shape loop and outer helix. The shorter VemP-2 covers residues S141–Q156, which span the inner helix and a part of the loop. The shortest VemP-3 spans only the inner helix and covers residues T146–Q156.

The initial structure of the ribosome-VemP complex was taken from the Protein Data Bank (PDB 5NWY) (17). It contains the *E. coli* ribosome, the P-site tRNA^Gln^ with a part of the wild-type VemP nascent chain (residues M120–Q156). Because all nucleotides are modeled as canonical nucleotides (A, C, G, U) in the cryo-EM structure, we added previously determined nucleotide modifications to the ribosomal RNA using the structure by Fischer *et al.* (PDB 5AFI) (37) as template. In the structure of the ribosome-VemP complex, no A-site tRNA was present. From this initial RNC complex, we prepared an alternative structure containing VemP-3 residues T146–Q156 in the ribosome tunnel. For each RNC, we prepared two initial conformations with either the A or B substate of U2506 (Figure 1A).

Each RNC complex was placed within a rhombic dodecahedron periodic box of water ensuring a minimal distance of 1.5 nm between the solute and the box surface. Structural K^+^, Mg^2 +^, Zn^2 +^ and Cl^−^ ions were taken from a previous cryo-EM structure (PDB 5AFI) (37) by superimposing the large and small ribosomal subunits separately. Excess ions were added at random positions in their respective concentrations according to cryo-EM experimental conditions (100 mM KCl, 10 mM MgCl_2_) and, subsequently, the whole box was neutralized by adding sufficient K^+^ ions. The resulting simulation box contained about 2.1 million atoms.

In addition, isolated VemP in aqueous environment was studied by MD simulations. For VemP-1, two initial conformations were used. First, the helical VemP conformation from the cryo-EM model was taken. Second, a non-helical extended conformation was used. For VemP-2 and VemP-3, only the helical initial conformations were used. The ion concentrations were identical to the RNC simulations.

The box size for MD simulations of VemP in solution was chosen such that the minimal distance between VemP and box surface was 2.7 nm. For instance, for VemP-1, this box size allows sampling conformations with an end-to-end distance shorter than 8 nm. Note that the end-to-end distance of the fully extended VemP-1 conformation is 13 nm. However, test simulations initiated from the fully extended conformation revealed that VemP collapses quickly (< 50 ns, Supplementary Figure S1) to a conformation with the diameter of about 3 nm, so using a smaller box to sample collapsed conformations is justified.

For simulations of the RNC, we used the Amber ff12SB force field (38) for the solute with the parameter set of non-canonical nucleotides based on ff99SB (39), the SPC/E water model (40), and Joung and Cheatham ion parameters (41). This setup has proven reliable in simulation studies of the ribosome (16,42). For VemP-1 in solution, we used two force-fields of different families. First, the identical force field as for the RNC complexes, namely ff12SB and SPC/E was used. Second, a recent force field CHARMM36m (43) was used, which was designed to better capture the equilibrium between folded and unfolded states. This protein force field was combined with the CHARMM-compatible TIP3P water model (44). Table 1 summarizes all simulated systems.

**Table 1.** Summary of MD simulation conditions

Simulation system	VemP	Sequence	Environment	Initial conf.	Force field	Trajs.	μs/traj
R1-AM-hel	VemP-1	M120–Q156	Ribosome	Helical	AMBER	2 x 4^a^	1.5
R3-AM-hel	VemP-3	T146–Q156	Ribosome	Helical	AMBER	2 x 4^a^	1.5
S1-CH-hel	VemP-1	M120–Q156	Water	Helical	CHARMM	8	4.0
S1-CH-ext	VemP-1	M120–Q156	Water	Extended	CHARMM	8	4.0
S1-AM-hel	VemP-1	M120–Q156	Water	Helical	AMBER	8	4.0
S1-AM-ext	VemP-1	M120–Q156	Water	Extended	AMBER	8	4.0
S2-AM-hel	VemP-2	S141–Q156	Water	Helical	AMBER	16	4.0
S3-AM-hel	VemP-3	T146–Q156	Water	Helical	AMBER	16	4.0

^a^ Four trajectories for each of the A or B substate of the U2506 nucleotide.

### Molecular dynamics simulations

Each simulation system listed in Table 1 was equilibrated in several steps. The solvent was energy-minimized keeping the solute fixed using Cartesian position restrains with a force constant of 1000 kJ mol^−1^ nm^−2^. Then, during 500 ps of MD simulation, the solvent was heated from an initial temperature of  10 K to 310 K using the v-rescale thermostat (45), while the solute was kept at 10 K. The inital velocities were selected randomly from a 10-K Maxwell–Boltzmann distribution. In the following 1 ns, the pressure (and the density of the simulated system) was equilibrated using the Berendsen barostat (46) targeted to 1 bar. Then the solute was heated to a temperature of 310 K while the position restraints were gradually released during another 20 ns MD simulation. Finally, production runs were carried out at 310 K and 1 bar (*in vivo* conditions (10,17)) using the v-rescale thermostat and the Parrinello-Rahman barostat (47), respectively.

In all simulations, the long-range electrostatic interactions were computed by the particle-mesh Ewald algorithm (48) with a 1.0 nm direct-space cut off, interpolation order of 4 and a 0.12 nm grid spacing. Van-der-Waals interactions were described by the Lennard-Jones potential with 1.0 nm cut off. All bond lengths were constrained by the LINCS algorithm (49). Hydrogens were described by virtual sites (50) to remove the fastest degrees of freedom, thus allowing for a time step of 4 fs (production runs).

All simulations were carried out using the GROMACS package (51) version 2016 (RNC complexes) and 2020 (VemP constructs in solution).

### Mimicking the conditions of the ribosomal exit tunnel

To assess the effect of the environment on the VemP SS, we performed CD measurements in mixtures of water and 2,2,2-triflouroethanol (TFE). TFE as co-solvent is known to stabilize protein SS, although this effect is typically levelling off around a 30–40 vol% range (52). The mechanism of TFE action is complex and consists of several competing factors (53–56). For instance, TFE displaces water in the solvation shell of proteins that promotes intramolecular hydrogen bonding. Also, TFE lowers ϵ_*r*_ and causes molecular crowding at high concentrations. It is also known that water/TFE mixtures show a viscosity η maximum around 30 vol% most likely due to TFE forming clusters which in turn results in more structured water (55).

To estimate ϵ_*r*_ of the exit tunnel, we considered that the average ϵ_*r*_ of proteins is 6–7 in the interior and 20–30 close to their surface (57). Based on experimental estimates of ϵ_*r*_ in desolvated condensed DNA of viral particles 6–10 (58) and of fully dissolved ribosomal RNA 70–80 (59), we assume that ϵ_*r*_ in the exit tunnel is likely higher than a typical protein surface, in the range 50–70.

Water/TFE mixtures emulate many of the estimated non-specific properties of the environment in the ribosomal exit tunnel, most importantly the reduced ϵ_*r*_ and increased η (60). The 20 vol% TFE mixture (ϵ_*r*_ = 69, η = 1.45 cP) provides a more bulk-like environment with a roughly 60% increase in η, whereas the 40 vol% TFE mixture (ϵ_*r*_ = 50, η = 1.78 cP) approximates more macromolecule-like solvent environment, similar to that of the exit tunnel near the PTC.

### Circular dichroism

VemP constructs (VemP-1, VemP-2 and VemP-3) were synthesized by Biomatik using solid state synthesis at a 95% purity level. The lyophilized peptides were verified by quantitative amino acid analysis and high-performance liquid chromatography. The peptides were dissolved in buffer solutions containing 50 mM sodium fluoride (NaF) and 10 mM sodium phosphate (NaPi) buffer (pH 7.2) as well as 0, 20 or 40 volumetric percent of TFE, respectively. Final peptide concentrations were determined by measuring UV absorbance spectra in the range of 200–350 nm, using the extinction coefficient at 280 nm, and ranged between 15 and 90 μM.

CD spectra of VemP constructs were measured at 25°C using a Jasco J-815 Circular Dichroism Spectropolarimeter. Spectra were recorded between 180 and 260 nm in 0.5 nm steps for both the peptides and the blank buffer solutions in 1 mm cuvettes. All peptide CD spectra were baseline corrected and normalized for protein concentration, cuvette path length, and amino acid numbers to compute mean residue ellipticities (deg cm^2^ dmol^−1^). All CD spectra were expressed in 1000 mean residue ellipticity units (kMRE). CD spectra were truncated to 190–260 nm wavelength range to ensure a linear concentration-dependent signal intensity at all remaining wavelengths. CD spectra were analyzed for secondary structure content as described below.

### Analyses

Structures of exit-tunnel walls and VemP seen in the MD simulations were characterized by residue-wise root-mean-square deviation (RMSD) calculated between MD conformations averaged over each trajectory and the conformations found in the cryo-EM model. Also for each VemP residue, we calculated root-mean-square fluctuation (RMSF), which reports on flexibility and mobility of each residue in the MD ensemble. Details are provided in the Supplementary Information.

MD-derived structural RNC models were obtained at three levels of averaging. First, as a mean structure over each of the individual trajectories, second, by averaging over all trajectories initiated from the same U2506 substate (A or B), and third, by averaging over all RNC trajectories. For the averages, only the latter parts of trajectories between 1.0 and 1.5 μs were used.

The SS of VemP was determined by using the DSSP algorithm (33). This algorithm defines eight SS motifs based on the overall geometry and hydrogen-bonding patterns. Helical content was calculated using the sum of α-helix and 3/10-helix propensities. All trajectories were processed by the GROMACS tool do_dssp (51).

The SS of VemP constructs were also determined from measured CD spectra using the Bayesian estimation module (SESCA_bayes.py) from the SESCA analysis package version 0.95 (61,62). SS estimates were obtained using the DSSP-TSC1 basis set, which contains three secondary structures and six side-chain correction basis spectra. Side-chain corrections were determined based on the AA composition of the corresponding VemP construct. The SS estimates were calculated from probability-weighted ensembles of 100 000 SS compositions generated by 100 Monte-Carlo chains started from randomly chosen secondary structures.

## RESULTS AND DISCUSSION

### Helical propensity of VemP sequence is weak

The reasons why VemP folds into the specific secondary structures of two helices (residues 120–130 and residues 146–156) and the S-shape loop (residues 131–145) in the ribosome exit tunnel are not clear. Su *et al.* speculated (17) that the PTC likely evolved ‘to generally disfavour excessive secondary structure formation’. The preference of VemP for α-helices may be encoded in its amino-acid sequence, it may be driven by the tunnel walls due to specific interactions between peptide and tunnel, in a nonspecific manner (e.g. by confinement, by the dielectric permittivity of the environment, etc.), or by a combination of these factors.

As a first test of the hypothesis that VemP sequence has an intrinsic helical propensity, we used two common SS prediction algorithms (Figure 1dD). The neural-network based three-state predictor JPred4 (34,35) classifies residues 122–134, which overlap with the residues of the outer helix, as helical (H), but only with a low confidence. In contrast, residues 148–153, which constitute the inner helix in the ribosome, are predicted as extended strand (E) with a high confidence. The NPS@ web server (36) yields a four-state prediction as consensus of 10 algorithms. For VemP, an extended strand (E) is predicted for residues 131–133 and 148–153, whereas the SS of the remaining residues is predicted to be unstructured.

Taken together, these SS predictions provide no evidence for strong sequence preferences of VemP to form the SS observed inside the ribosome.

### CD spectroscopy shows that VemP in water is disordered

To estimate SS preferences of VemP in different environments, we carried out CD measurements of three VemP constructs whose sequences are denoted by VemP-1, VemP-2, and VemP-3 in water as well as in water/TFE mixtures and analyzed them by SESCA (61,62). VemP-1 refers to the whole peptide resolved by cryo-EM, VemP-2 comprises the inner helix with a part of the S-shape loop, and VemP-3 contains the inner helix only (Figure 1c, see Materials and Methods). Figure 1E shows the SS preferences of VemP in various solvents. Three-state SS classification was used to differentiate between helix, strand, and coil. The spectra of all measurements are shown in Supplementary Figure S2.

In water, none of the VemP constructs showed a sizeable helical content (1–3 %, Figure 1E). In fact, each VemP construct appeared to be about 80% disordered, and partially adopted extended β-strand-like conformations. Within the experimental uncertainty of 3–4 percentage points, no differences were observed in the helical content of the three VemP constructs in water.

CD measurements in 20 vol% water/TFE mixture indicated an increased helical content for all three VemP constructs: The estimated helical content is about 25% for VemP-1, 12% for VemP-2 and 22% for VemP-3. Further addition of TFE to 40 vol% led only to a small increase of the helical content of VemP-1 (26%) and VemP-3 (24%), but a considerable increase to 36% for VemP-2. Overall, the measured helical contents were markedly lower than those calculated as the ratio of helical and non-helical AAs from the cryo-EM model (Figure 1d), namely 38% for VemP-1, 50% for VemP-2 and about 80% for VemP-3. These differences in helical contents imply that the structure of VemP in water and in ribosome are also different, which agrees with the conclusion drawn from the sequence-based SS predictions.

Different helical contents of VemP-1 in different water/TFE mixtures also suggest that non-specific interactions with the solvent play a significant role in the SS formation of VemP. Further, the lower helix content of VemP-1 in TFE (26%) compared to cryo-EM (38%) suggests that further SS stabilizing factors contribute to the increase helicity in the ribosomal tunnel, with steric restriction and specific interactions with the tunnel wall being likely candidates.

The helical content of VemP-3 in 40 vol% TFE (24%) is much lower than the cryo-EM estimate (80%), approximately half of what we would expect based on VemP-1 and VemP-2 comparisons. This observation indicates stabilizing intra-molecular interactions between the inner helix of VemP and the loop region present in VemP-1 and VemP-2 but not in VemP-3.

It might be possible that further increase of TFE concentration would lead to higher helicity in VemP constructs, leading to a better agreement with the cryo-EM structure. However, we consider this unlikely as the helical content of both VemP-1 and VemP-3 appear to saturate as a function of TFE concentration, and TFE concentration of more than 40 vol% often have a destabilizing effect on peptide secondary structures (52), thus corroborating the presence of further stabilizing factors within the tunnel.

### Helicity in simulations compared to experiments

To validate our simulation approach, we checked if the SS observed in the simulations agrees with the SS observed by cryo-EM in the ribosome and the SS obtained from CD measurements in water. Helical content of the peptides and SS propensities of individual AAs were calculated from simulation trajectories using the DSSP algorithm (33), where an AA was considered helical if it was classified as α-helix or 3/10-helix. All SS classifications as function of simulation time are shown in Supplementary Figures S3–S5.

**Figure 2.**
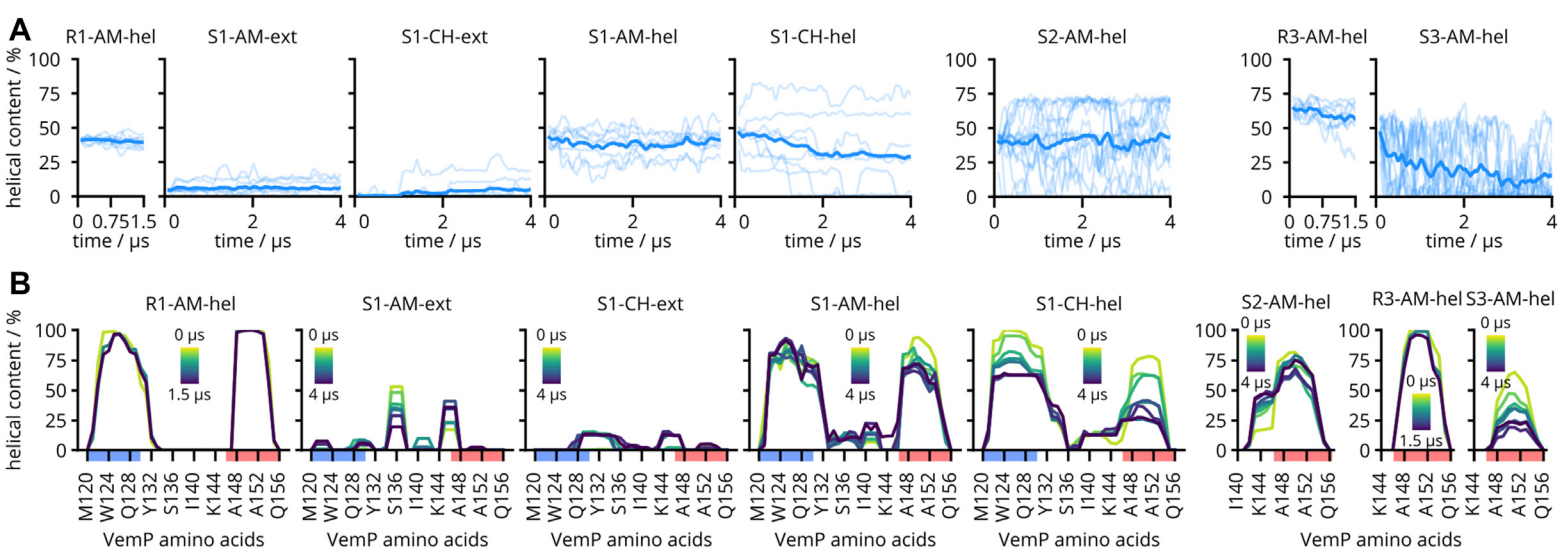
VemP helicity obtained from MD trajectories using DSSP (33). Plot titles refer to in-ribosome (R) or in-solvent (S) simulations, of VemP-1, 2 or 3, using AMBER (AM) or CHARMM (CH) force field, initiated from the helical (hel) or non-helical extended (ext) VemP conformation. (**A**) Helical content of the peptide as a function of simulation time. Individual trajectories are shown as thin lines, the average over the trajectories is in bold. For clarity, all lines represent running averages over 100 ns. (**B**) Helical content of individual AAs averaged over time. Several time blocks color-coded from yellow (beginning of simulations) to navy (end of simulations) are averaged over independent trajectories. Lines are separated in time by 500 ns; longer simulations resulted in more lines. Sequence regions classified as helical in the cryo-EM structure are highlighted in red (inner helix) and blue (outer helix) on the horizontal axis.

In the ribosome, both helices of VemP-1 remained fully helical during all eight 1.5-μs simulations and close to their conformation in the cryo-EM structure, which is shown by the backbone RMSD lower than 0.1 nm (Supplementary Figure S6). Also the peptide helical content was about 45% and remained constant throughout the simulations (Figure 2A, left panel). VemP regions with high residue-wise helical content calculated from the MD simulations of VemP in the ribosome agree well with the regions classified as helical in the cryo-EM structure (Figure 2B, left panel).

Simulations of VemP-1 in water were started from a fully extended conformation using two force fields and eight independent simulations, 4 μs-long each. Peptides in these simulations remained mostly unfolded, although transient helix formation was observed for both force fields. The helical content varied between individual simulations showing that they are not fully converged (Figure 2A, B). However, the helical content of VemP-1 averaged over all trajectories, which is $6 \pm 5\%$ for the AMBER force field (39) and $3 \pm 5 \%$ for the CHARMM force field (43), agrees with the estimated helix content of $2 \pm 3 \%$ obtained from CD spectroscopy (Figure 1E).

The low helical content of VemP-1 in water observed in CD measurements and in simulations suggest that the high-helicity ribosome-bound conformation is unstable outside the ribosome. However, a small population of this conformation cannot be ruled out by the low but non-zero helical content and the MD simulations. To directly check if the ribosome-bound conformation corresponds to a local free-energy minimum, we performed MD simulations of the three VemP constructs in water starting from their ribosome-bound conformations.

For VemP-1 the initial helix content decreased in CHARMM force field simulations from 50% to about 25% on average after 4 μs (Figure 2A). The decrease of helicity was faster for the inner helix than for the outer helix (Figure 2B). In the AMBER force field simulations, the unfolding kinetics appear to be slower, and mostly limited to the inner helix for which the helicity was reduced from almost 100% to about 75%. Here, the decrease of the inner helix content was compensated by an increased helical content of residues 132–144 in the S-loop region, resulting in a roughly constant helical content of 45% for the entire peptide in AMBER.

As expected, the helical content did not converge on the timescales of the simulations. However, the observed rapid decrease in helicity of the inner helix on these short timescales suggests that the ribosome-bound conformation of VemP does not correspond to a metastable conformation in water. Taken together, the above results strongly suggest that the environment in the ribosome stabilizes the stalling conformation.

The trends observed for VemP-2 are very similar to VemP-1, with helicity decrease in the inner helix region and increase in the S-loop region. However, the helicity of individual VemP-2 trajectories varied greatly from 75% to nearly 0% which may indicate a cooperative metastable helix structure spanning most of the VemP-2 sequence.

VemP-2 adapted two extreme conformational states in the simulations, a collapsed state with a hydrophobic core formed around W143 and a fully helical state, in which a helix spans the entire VemP-2 sequence (Supplementary Figure S4). These two states may also explain the unexpected helical content obtained by CD spectroscopy. If the collapsed state corresponds to the free-energy minimum of the VemP-2 in water, it would result in a very low helix content. It is conceivable that in the more hydrophobic 20 vol% water/TFE mixture, this minimum would still be present, but more helical conformations would become energetically more favorable, resulting in the lower helical content. Increasing the TFE concentration to 40 vol% might then result in the helical state becoming the free-energy minimum leading to the measured high helix propensity (Figure 1E).

From the three VemP constructs, VemP-3 (inner helix only) showed the largest decrease in helicity, dropping from 50% to 10%. Within the 4-μs trajectories multiple folding and unfolding events were observed for VemP-3 (Supplementary Figure S4), suggesting a transient helix behavior as discussed for the VemP-1 simulations.

In summary, these results show that the simulations are able to capture the SS composition observed in cryo-EM (17) and in CD measurements.

### Stalling VemP conformation is stabilized by intramolecular interactions and interactions with the ribosome

To assess the role that the N-terminal VemP parts (S-loop and outer helix) play in the stalling mechanism, we also simulated VemP-3 in the ribosome tunnel. These 1.5 μs-long trajectories showed a decrease of the average helical content from about 65% to 55% on average, and in one of the eight trajectories the helical content decreased to 25%. The backbone RMSDs were larger compared to the VemP-1 simulations (Supplementary Figure S7).

The largest change of average helicity in these simulations was observed for N-terminal residues T146 and N147. Note that in the cryo-EM structure, E145 forms two hydrogen bonds with the S-loop residues H138 and R139. These stabilizing interactions are absent in VemP-3. The loss of helicity of VemP-3 in the ribosome exit tunnel suggests that the S-loop stabilizes the inner VemP helix within the tunnel.

Even though our simulations are not long enough to describe the full secondary structure dynamics of VemP in a quantitative manner, they collectively allow us to draw several conclusions.

First, the simulations showed a very stable helix-loop-helix structure of VemP within the ribosome and VemP is primarily disordered in water, in agreement with the cryo-EM data and our CD spectroscopy measurements. Second, the folding dynamics of VemP in water happens on timescales longer than μs, and the inner helix region of VemP is particularly unstable. Apparently, the inner VemP helix requires both intramolecular interactions with the S-loop and intermolecular interactions with the ribosomal exit tunnel. Adding a co-solvent like TFE mimics some of the non-specific interactions of the ribosomal environment, but does not suffice to reach the helicity level observed in the tunnel. Therefore, specific interactions between VemP and the tunnel walls are likely necessary for the inner helix to be fully folded.

Interestingly, in the simulations of VemP-1 initiated from the extended conformation, the largest helicity was observed for residues 128–136 and 144–148, which corresponds to the helical regions flanking the S-loop in the ribosome-bound conformation. We speculate that, in the ribosome, folding of the helices required for stalling starts at these residues and progresses towards the N-terminus for the outer helix, and towards the C-terminus for the inner helix. Since these results suggest that the interactions between the peptide and the exit tunnel are crucial for stalling, we will subsequently focus on the atomistic details of these interactions and the dynamics of VemP within the tunnel.

### Validation of ribosome-bound VemP simulations

To investigate the key interactions between VemP and the ribosome tunnel wall, we first assessed the quality of the MD simulations of VemP-1 in the ribosome, by comparing the average structure obtained from MD trajectories with the cryo-EM model. For this comparison, MD simulation frames were aligned to the cryo-EM model using a least-square fit of the ribosome tunnel atoms. Thus deviations between the two models represent the changes in VemP position within the tunnel, as well as internal conformational changes of VemP.

**Figure 3.**
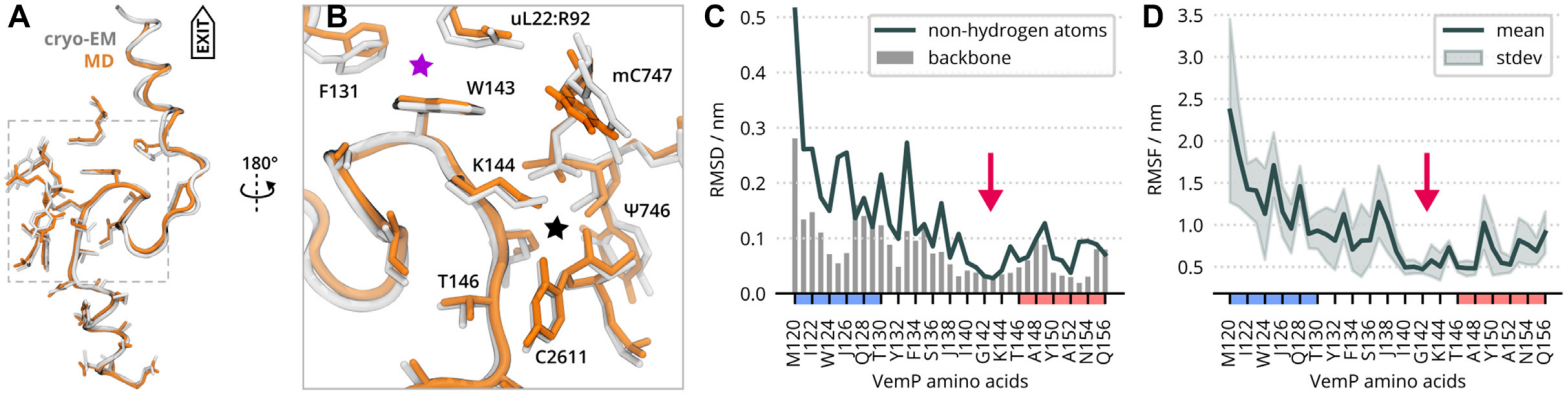
MD-generated model of ribosome-bound VemP. (**A**) The MD-generated model (in orange) was obtained as an ensemble average of all RNC trajectories (U2506 substates A and B). The cryo-EM model is in gray. (**B**) Context of the structurally conserved VemP part. W143 stacks to R92 of ribosomal protein uL22 and interacts with F131 of VemP (magenta star). The K144 side chain directs to a pocket formed by the phosphate between pseudouridine Ψ746 and 5-methylcytosine mC747 and with oxygen of C2611 (black star). (**C**) Residue-wise RMSD between cryo-EM and MD models. The black line represents the RMSD of non-hydrogen atoms, blue bars stand for the RMSD of the VemP backbone. The red arrow indicates structurally conserved region of VemP – the anchor. Sequence regions classified as helical in the cryo-EM structure are highlighted in red (inner helix) and blue (outer helix) on the horizontal axis. (**D**) Residue-wise root-mean-square fluctuation (RMSF). The mean value over eight independent RNC trajectories is shown as line, the shaded area represents the standard deviation. Sequence regions classified as helical in the cryo-EM are highlighted in red (inner helix) and blue (outer helix) on the horizontal axis.

Superposition of the average MD structure and the cryo-EM model depicted in Figure 3A shows that our ribosomal VemP simulations not only maintained the SS, but also the position of VemP in the tunnel and side chain orientations for the inner helix and loop structures. This close agreement between the average structure and the cryo-EM models is quantified by a 0.09 nm average deviation (RMSD) of the VemP-1 backbone atoms.

The residue-wise RMSDs depicted in Figure 3B show how the deviation from the cryo-EM model changes along the VemP-1 sequence. The RMSD of the inner-helix and S-loop backbone atoms is only around 0.05 nm for residues 139–156. For outer helix residues, the backbone RMSD is notably larger and increases with increasing distance from the PTC, up to 0.29 nm at M120.

The increase of N-terminal RMSDs is probably caused by the truncation of full VemP to VemP-1 in the simulations. In the cryo-EM experiments, a longer construct was used including residues 26–119 (17). However, this part was not resolved in the final cryo-EM model presumably because of its higher flexibility. The truncation likely increased the flexibility of the N-terminal residues and increased RMSDs, similar to the effect observed upon truncation of VemP-1 to VemP-3 (Supplementary Figure S8). However, these deviations are relatively small, and, as Figure 3B shows, they did not affect the S-loop region or the inner helix regions.

In summary, the RNC simulations closely resemble the VemP conformation near the ribosome catalytic center, and the deviation from the cryo-EM model of the backbone RMSD remained below 0.14 nm for both VemP helices during the simulations (Supplementary Figure S6). These results strongly suggest that the RNC simulations are consistent with the experimental information about the structure of the stalling competent VemP-ribosome complex, and allow us to proceed with the analysis of key interactions between VemP and the tunnel wall.

### Structurally conserved loop region may act as anchor

To characterize VemP dynamics, we calculated the residue-wise RMSF of the aligned MD trajectories (Figure 3C). The RMSFs quantify the mobility of VemP residues relative to the tunnel wall. Regions with low RMSF indicate regions that form strong interactions with the tunnel wall. Intriguingly, the region between residues I140 and K144, or even up to N147, is structurally very well conserved, i.e. shows both small RMSFs and small RMSDs between MD simulation and cryo-EM (red arrows in Figure 3C and D). This region is located at the N-terminus of the inner helix near the constriction site. It includes residues W143 and K144, which were previously identified as critical for stalling (10,23). In our simulations, the positively charged side chain of K144 interacted with the negatively charged phosphate of mC747 (black star symbol), whereas W143 forms a contact with R92 of the constriction site protein uL22 and F131 of VemP (purple star symbol).

A biochemical characterization of VemP previously reported that the interactions of K144 and mC747 appear to be crucial, as any mutation of K144, apart from positively charged AAs (Arg, His), alleviated stalling (23). The interactions observed for W143 are also in line with the suggestions of Su *et al.* (17), that the outer helix plays a role in translation arrest by possibly stabilizing this VemP region.

It is conceivable that during the incremental elongation of NCs, the identified residues act as an *anchor*, that slows down the VemP progress through the tunnel to enable α-helix formation. A similar mechanism was recently reported by Herrero del Valle et al. in the context of ornithine sensing by ribosomes (7). In their structure, a NC sensing domain binds the tunnel around U464, which is located about 4 nm away from the PTC. For VemP, anchoring would occur about in a distance of 3 nm from the PTC, around the ribosomal residues Ψ746 and C2611 (Figure 3B). Besides affecting the kinetics, the anchor may also stabilize the transient helices suggested by our extended VemP-1 simulations and CD measurements in water. It is possible that initially the helical structures correspond to a shallow free energy minimum, which becomes gradually deeper for subsequent VemP intermediates, due to increasing confinement and additional interactions with the tunnel walls. In this scenario, the N-terminal α-helix eventually becomes the deepest minimum on the free energy landscape, such that the helix inhibits further peptide bond formation.

### Analysis of U2506 substates uncovers substates also of the VemP inner helix

The cryo-EM model represents the average structure of substates A and B with the exception of U2506, where the cryo-EM model shows two different substates (17). In contrast, MD simulations should allow to resolve the substates of the whole RNC conformation, possibly also depending on the initial substate of U2506.

**Figure 4.**
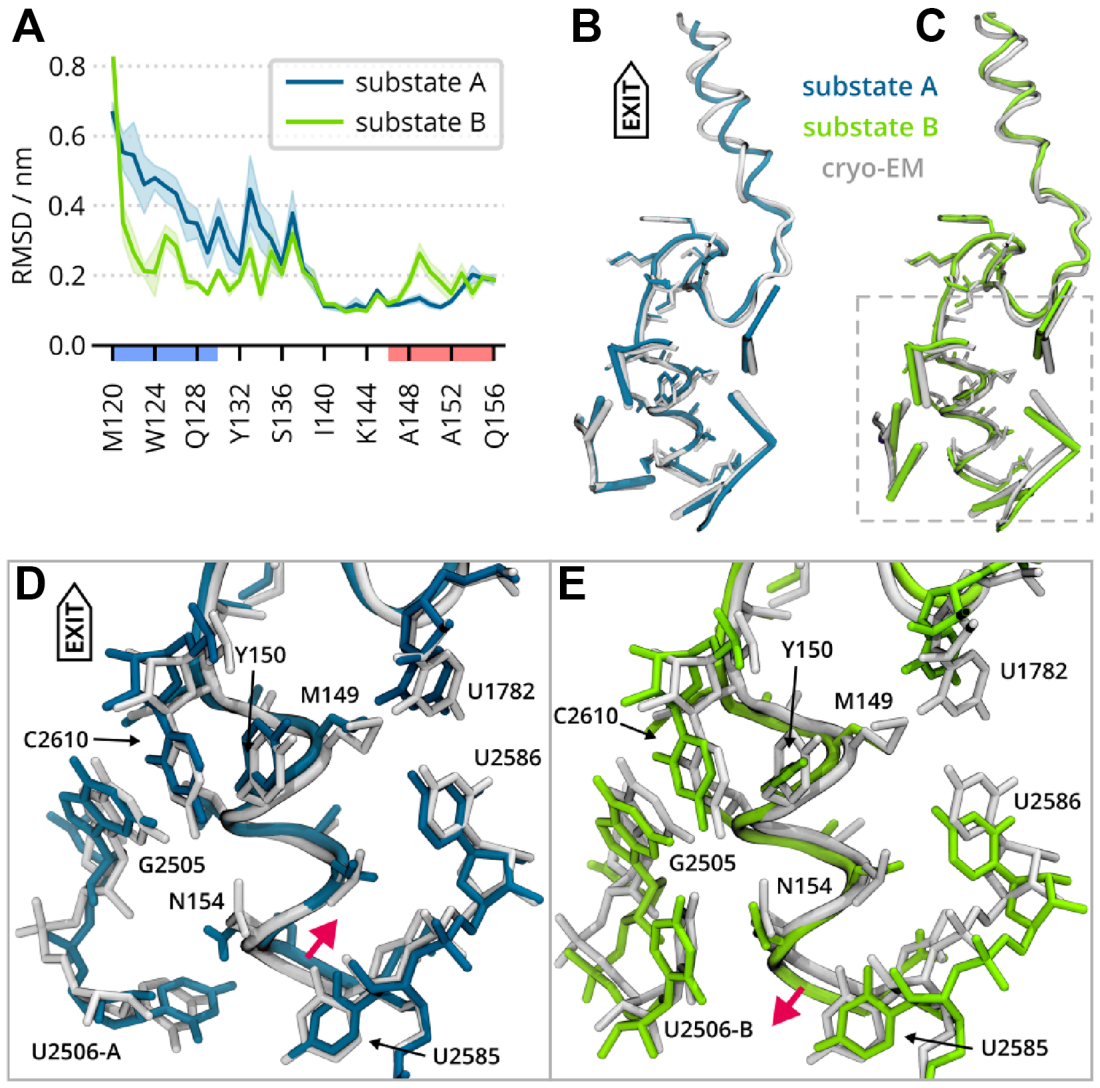
Structural differences resulting from U2506 substates. (**A**) Residue-wise RMSD between the cryo-EM model and structures averaged over all the trajectories initiated from each substate. The mean RMSD value over the 4 independent trajectories is shown as a line, the shaded area represents the standard error of the mean. On the horizontal axis, the AAs of the inner or outer helix are highlighted in red and blue, respectively. (**B**) Comparison of the structures averaged over all trajectories initiated from U2506 in substate A (in teal) and the cryo-EM model (in gray). Selected ribosomal nucleotides are represented as sticks, whereas the side chains of residues 143–156 are shown as licorice. (**C**) Same as b) but for the substate B. (**D**) The detail of the VemP inner helix and its surroundings as framed in C), but depicted as licorice. The MD-derived model from trajectories initiated from the substate A is is in teal, cryo-EM in gray. Red arrow indicates a dislocation of the helix with respect to the cryo-EM model. (**E**) Same as d) but for the substate B.

Indeed, Figure 4 shows clear differences between simulations initiated from substates A and B. These differences become evident in Figure 4A, which shows the residue-wise RMSDs of non-hydrogen atoms of VemP. These were calculated for each of the VemP residues for the structural models obtained as averages over MD trajectories initiated either from substate A (Figure 4B) or substate B (Figure 4C) of the U2506 with respect to the cryo-EM. Figure 4D and E shows the context of the inner VemP helix in the ribosome tunnel for substates A and B, respectively.

In the RNC simulations, we observed two distinct conformational ensembles of VemP and its neighborhood for the substates A and B. During the 1.5 μs simulations, no transition between the U2506 substates occurred. Differences between the ensembles of substates A and B were found up to 2 nm away from the C-terminus of VemP, or to a distance of roughly three nucleotides from the U2506. These differences are remarkable because the RMSDs of the structure obtained from averaging over the trajectories of both substates are much smaller, which shows that the cryo-EM structure is indeed an average of two conformational states of the ribosome-VemP complex that do not only differ with respect to the U2506 conformation.

Notably, in both substates, the position of the VemP C-terminal residues Y150–Q156 differed (red arrows in Figure 4D and E). In substate A, U2506 partially occupies the ribosomal A site, and it pushed the helix towards the tunnel exit more than in substate B. There was also an interaction between N154 and U2506 in substate B (but not in A) that can influence the position of the VemP C-terminus. Average positions of the C-terminal Q156 in the direction of the exit tunnel differed by about 0.15 nm between the two substates.

The residue-wise RMSDs have two main features: First, the N-terminal part up to H138 has a higher RMSD in substate A than in B, showing that the structures of the outer helix found in the four independent trajectories deviated from the cryo-EM model more in substate A than B. Second, a part of the inner helix (A148–A152) had larger RMSD values in substate B than in A. Structural variations were observed for AA side chains rather than the VemP backbone. The largest difference was found for M149 with a distance of 1.1 nm from the VemP C-terminus. The U2506 substate affected the vicinity of M149, namely the hydrogen bonding between U1782 and U2586, and stacking of Y150 with C2610. The former interaction was affected by U2585, whereas the latter is affected by G2505 (Figure 4D, E).

### The inner VemP helix is stabilized in the ribosome tunnel

To assess the role that the N-terminal VemP parts (S-shape loop and outer helix) play in the stalling mechanism, we simulated VemP-3 (inner helix) in the ribosome tunnel.

In solution, the helical content of VemP-3 was very low in both CD spectroscopy and simulations, so it is conceivable that the helix may also unfold in the tunnel without being held in place by anchor and loop. Because the helix sterically prevents U2506 and U2585 from adopting translation competent conformations, unfolding of the helix could release the stalling.

**Figure 5.**
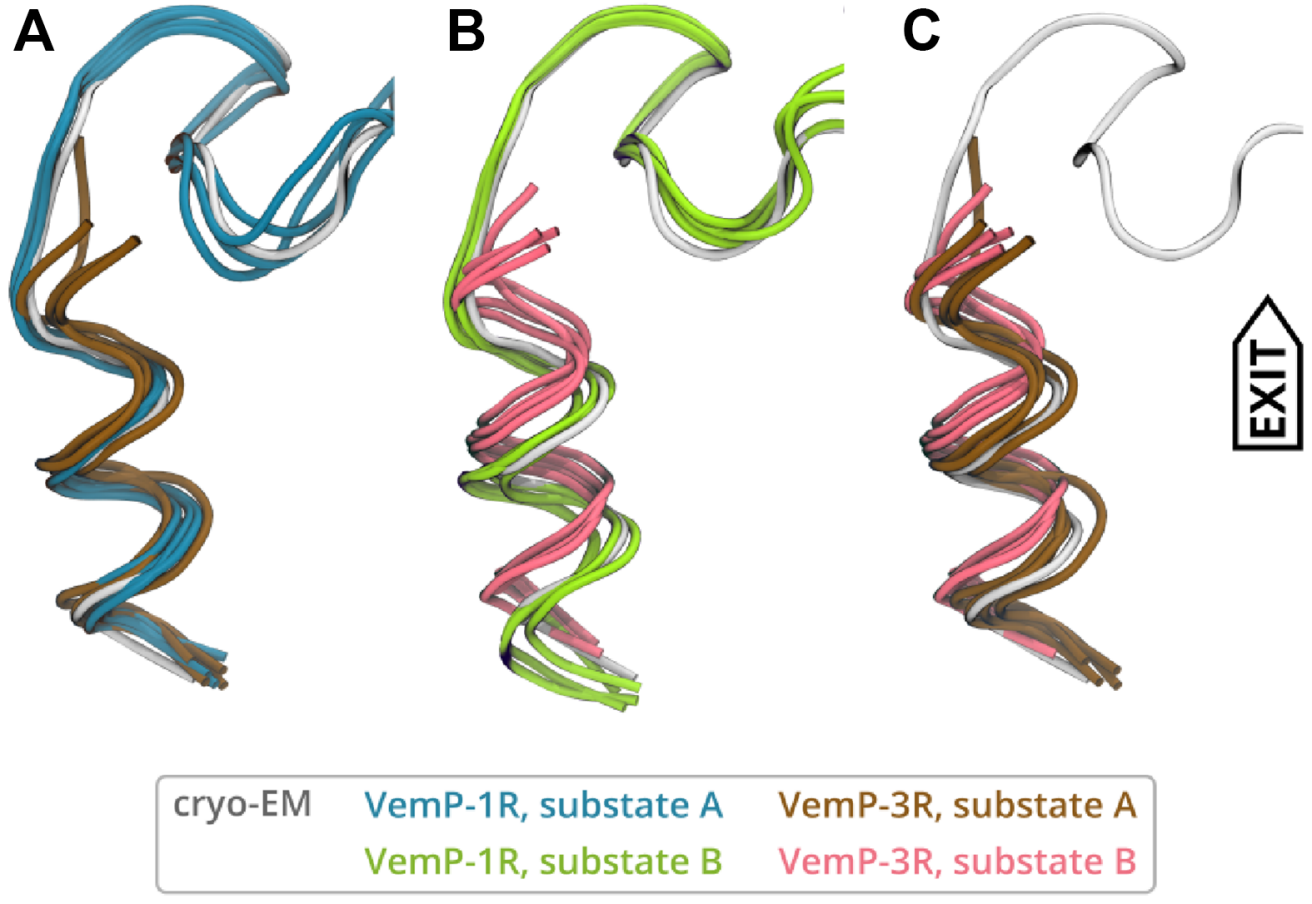
Comparison of VemP-1 and VemP-3 in substates A (**A**), and B (**B**) in the ribosomal tunnel. VemP-3 substates are compared in (**C**). The cryo-EM model is shown in gray for reference. Each structure represents the mean conformation of one of the four independent trajectories.

There is a notable difference in the VemP-3 helix position relative to the tunnel between the substates A and B. Whereas the inner helix stayed in place in substate A, in substate B, the entire helix shifts by about 0.15 nm towards the tunnel exit (Figure 5). The observed VemP-3 shift resembles a spring release triggered by *in-silico* removal of the large N-terminal part. This suggests that the stalled VemP peptide is under strain in the narrow space near PTC and that the strain is more pronounced when U2506 resides in state B, where it directs towards the tunnel exit and further reduces the diameter of the tunnel near the PTC.

Nucleotides surrounding the helix C-terminus adopted similar conformations as in the VemP-1 case. U2506 and U2585 stayed unaffected by the missing S-shape loop and the C-terminal helix in VemP-3 simulations in the ribosome. The nucleotides closer to the constriction site, which originally resided near the removed VemP part, changed their structure and dynamics. For instance, U1782 and U2609 were more flexible compared to the case of VemP-1.

We expect VemP-3 to be stalling incompetent, although no biochemical characterization of VemP-3 has been done so far. It was reported that a slightly longer VemP construct with residues 138–156 showed a stalling efficiency reduced to 10% compared to the full-length VemP (10,17). Even though we did not see a complete unfolding of the inner helix on the time scale of the simulations, the reduced helical content and the shift of the helix might be the first step towards the release of the U2506 and U2585. The faster and more complete unfolding observed in the simulations of VemP-3 in solution suggests that the interactions between the helix and tunnel constitute a free-energy barrier for helix unfolding.

## CONCLUSION

Nascent chains can stall ribosomal translation by various mechanisms (1) including inhibition of peptide-bond formation (10), tRNA translocation (63), and peptide release (5). A wealth of structural information on the conformations of stalling peptides bound to the ribosome lead to the identification of the molecular mechanisms underlying the inhibition of these steps of translation. However, general principles of how nascent chains adopt stalling conformations remain unclear. Here we studied the molecular details of translational arrest of the VemP peptide, which folds into two compact helices in the ribosomal exit tunnel and interferes with tRNA accommodation.

The sequence of VemP and even of its inner helix is unique and, as for now, it has been found only in the genus Vibrio. Thus it seems to be evolutionary highly optimized for its purpose (23). Still, the sequence itself lacks strong helical propensity in water, as we have shown by CD spectroscopy and MD simulations.

We found that the conformations and dynamics of VemP within the ribosomal tunnel are very different compared to VemP in solution showing that it is largely determined by the environment. We found that both the non-specific properties of the environment as well as specific interactions between conserved residues of VemP and the environment contribute to the stability of the α-helix.

**Figure 6.**
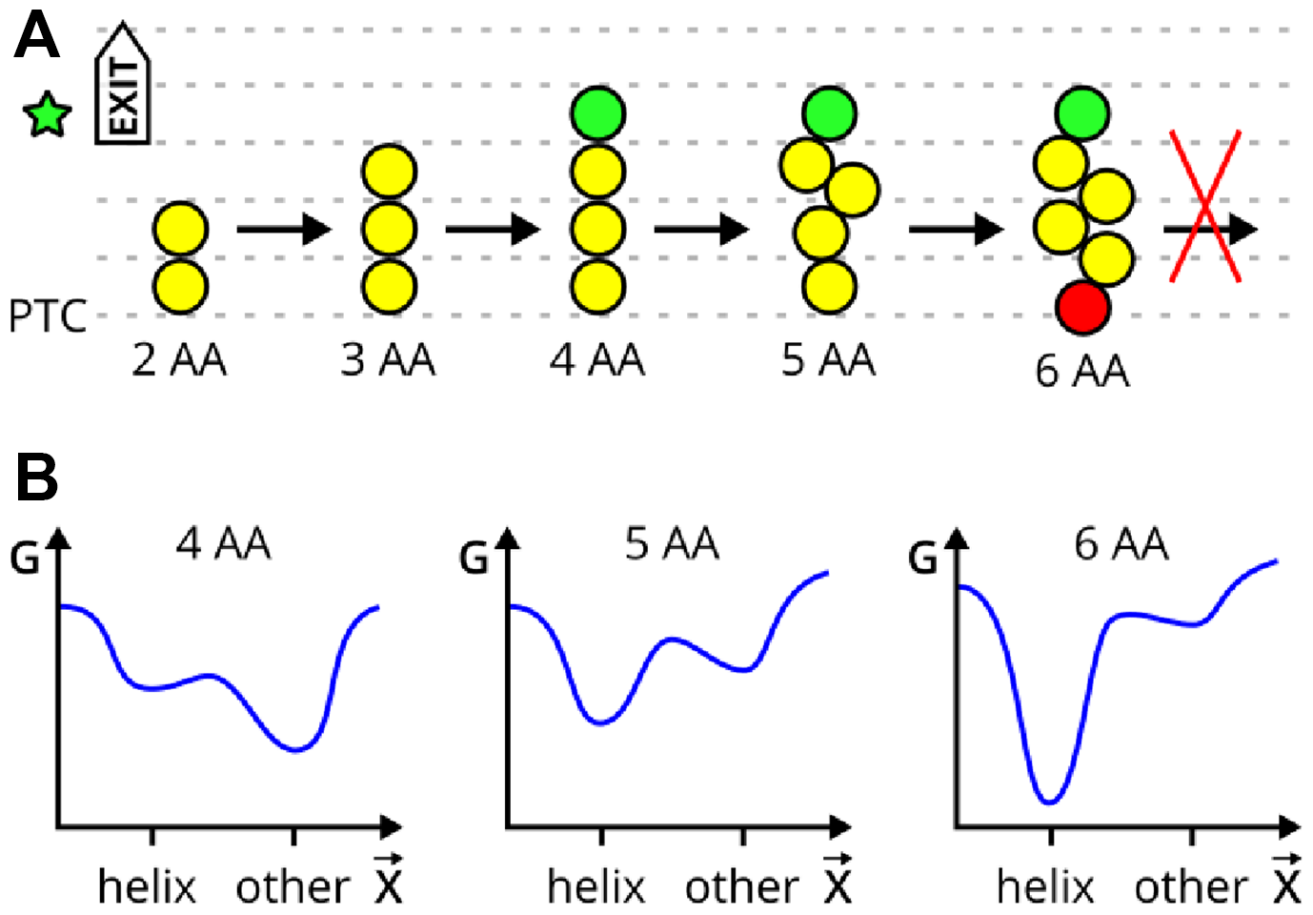
A model for anochor-mediated VemP translational arrest. (**A**) Nascent peptide (NP) elongation proceeds until the anchoring amino acid (AA, green circle) reaches the anchoring point of the tunnel (green star). The anchoring interactions prevent the NP from translocating further, such that secondary structure content increases upstream until a sterical clash with the PTC (red circle) occurs, ultimately causing arrest. For simplicity, less AAs (yellow circles) than present in VemP is shown. (**B**) Sketch of a possible conformational free energy (G) landscapes of VemP intermediates in the tunnel with respect to a generalized conformation coordinate $\vec{X}$ that characterizes NP translocation. The landscapes corresponds to NP from (A) of 4, 5 and 6 AAs.

Additionally, detailed analyses of our MD simulations of two U2506 substates revealed a region of VemP that is structurally conserved and rigid. The results suggest two distinct roles which VemP AAs play during translation arrest: The inner helix (residues 146–156) clearly interferes with the two critical nucleotides (17), but it needs about 10 VemP AAs (I135–E145) with several specific and possibly strong interactions around W143 and K144 to form and to become stable and well-localized within the tunnel. We argue that these residues serve as an anchor, which suggests a simple model explaining the sequence dependent formation of the inner VemP helix and the subsequent translational arrest (Figure 6).

In this model, we assume that the rate of AA addition (in bacteria several AA per second) is much slower than the relaxation times and folding dynamics of the VemP peptide within the tunnel. Therefore, during each elongation step (snapshots in Figure 6), the VemP peptide reaches local conformational equilibrium. VemP intermediates translocate through the tunnel until the anchoring AAs interact with the tunnel walls (Figure 6A). This interaction changes the conformational free-energy landscape of subsequent VemP intermediates (Figure 6B). Our CD measurements suggest that the free-energy minimum corresponding to the α-helix is initially shallow and is determined by a non-zero sequence helical propensity in a hydrophobic environment. Upon every AA addition, the minimum becomes deeper due to specific interactions of N-terminal VemP residues and tunnel walls, as determined by cryo-EM (17) and observed in our MD simulations.

It remains unclear whether the outer VemP helix is formed before or after the anchoring interactions are formed. Nevertheless, at least through the F131-W143 contact, there is a mutual stabilization of the outer helix and the anchor, that could also explain the previously observed dependence of stalling efficiency on the presence of the outer helix. As argued in a study of hydrophobic and hydrophilic α-helices, the accommodation of helical NCs in the ribosome tunnel might be a common phenomenon (27).

The anchor-mediated translational arrest represents an allosteric mechanism where a local change of the interaction between AAs and the anchor site causes a distant effect at the PTC. In fact, similar allosteric effects onto the PTC were reported for other arrest peptides such as ErmCL (16) or SecM (64). Notably, the allostery through various compacted motifs of non-arresting NCs was also demonstrated in the opposite direction towards the tunnel exit (28).

The allostery studied here, however, differs from the classic view, where a conformational change within a *single* biomolecule or biomolecular complex is responsible for the signal transduction (65). In our model, the signal is transferred through a modification of free-energy landscapes of *multiple* chemical reactions involving VemP intermediates.

In terms of reaction kinetics, the assumption that each VemP intermediate reaches conformational equilibrium implies that the rate of peptide bond formation is much lower than the rate of NC translocation through the tunnel. This would suggest that the rate of NC translocation may be essential for the stalling mechanism, in line with accumulated evidence that the rate of elongation has profound regulatory consequences (66).

Our conclusion that VemP requires specific interactions with the tunnel walls to acquire the secondary structure can also be cast in terms of the principles of protein folding outside the ribosome. Folding of cytosolic proteins is initiated by long-range tertiary contacts (67) that reduce the conformational space of the protein. Tertiary contacts were shown to be essential for α-helix formation (68,69). Here, the ribosome tunnel could provide these contacts through the anchoring interactions to promote helix formation.

## Supplementary Material

gkac038_Supplemental_File
