# Supplementary material for: Folding of VemP into translation-arresting secondary structure is driven by the ribosome exit tunnel
Source: Nucleic Acids Res. 2022 Feb 12;50(4):2258–69. doi: 10.1093/nar/gkac038 (PMC8887479; doi:10.1093/nar/gkac038)
Supplement: gkac038_Supplemental_File [file gkac038_supplemental_file.pdf]

Supplementary Information to "Folding of VemP into translation-arresting secondary structure is driven by the ribosome exit tunnel"

Michal H. Kolář,[*,†,¶] Gabor Nagy,[†] John Kunkel,[‡] Sara M. Vaiana,[‡] Lars V. Bock,[†] and Helmut Grubmüller[*,†]

†*Max Planck Institute for Biophysical Chemistry, Am Fassberg 11, 37077 Göttingen, Germany*

‡*Department of Physics and Center for Biological Physics, Arizona State University, 85287 Tempe, AZ, USA*

¶*University of Chemistry and Technology, Technicka 5, 16628 Prague, Czech Republic*

E-mail: michal@mhko.science; hgrubmu@gwdg.de

Analyses

Residue-Wise Root-Mean-Square Deviation

The trajectories were superimposed onto the cryo-EM model using the backbone atoms of the exit tunnel (Tab. S1). The average structures were calculated for 1000–1500 ns of each of the trajectories. The $\mathrm{RMSD}_{s,t}$ of a residue s between conformation in the trajectory t and the cryo-EM model was calculated as

$$\mathrm{RMSD}_{s,t} = \sqrt{\frac{1}{N_s}\sum_{i}^{N_s} |\mathbf{r}_{s,t,i}(\mathrm{MD}) - \mathbf{r}_{s,i}(\mathrm{cryoEM})|^2}, \tag{1}$$

where $\mathbf{r_{s,t,i}}$ is the mean value of the position vector of the atom i in residue s over the portion of trajectory t, $\mathbf{r}_{s,i}$ if the position vector of the atom i in residue s in the cryo-EM model, $||$ stand for the norm of a vector, and N_s is the number of atoms of residue s.

The RMSD of a residue s was then calculated as the mean value of $\mathrm{RMSD}_{s,t}$ of the trajectories.

Root-Mean-Square Fluctuations

The root-mean-square fluctuation of the residue s over the trajectory t was calculated as

$$\mathrm{RMSF}_{s,t} = \sqrt{\frac{1}{N_{t,f}} \frac{1}{N_{s,i}} \sum_{i}^{N_{s,i}} \sum_{f}^{N_{t,f}} \left(\mathbf{r}_{i,f} - \langle \mathbf{r}_{i,f} \rangle\right)^2 +} \tag{2}$$

where $\mathbf{r}_{i,f}$ is the position vector of atom i in the trajectory frame f, the brackets $\langle\,\rangle$ stand for the mean value, $N_{t,f}$ is the number of frames of trajectory t and $N_{s,i}$ is the number of atoms in the residue s.

The RMSF of a residue s was then calculated as the mean value of $\mathrm{RMSF}_{s,t}$ of the trajectories. The RMSFs were calculated for the latter parts of trajectories between 1000 and 1500 ns.

Tables

Table S1: Numbers of the residues used for the least-square alignment of MD trajectories (*E. coli* numbering).

23S rRNA 746 747 748 751 752 789 790 1614 1781 1782 2057 2058 2059 2061 2062 2063 2064 2251 2252 2253 2439 2450 2451 2452 2453 2503 2504 2505 2506 2507 2508 2553 2554 2555 2573 2576 2581 2583 2584 2585 2586 2601 2602 2608 2609 2610 2611 2612

AA-tRNA 73 74 75 76

protein uL4 55 56 57 58 59 60 61 62 63 64 65 66 67 68 69 70 71 71 73 74 75

protein uL22 80 81 82 83 84 85 86 87 88 89 90 91 92 93 94 95 96 97 98 99 100

Figures

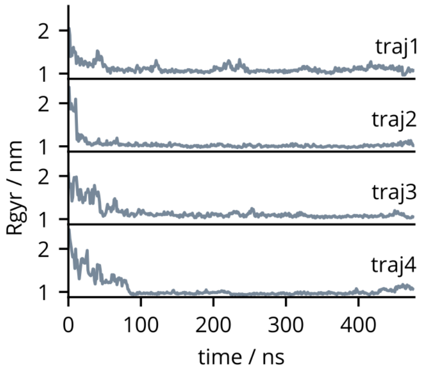

Figure S1: Radius of gyration of VemP-1 in solution (i.e. out of the ribosome) as a function of simulation time. The simulations were started from the fully extended VemP conformation.

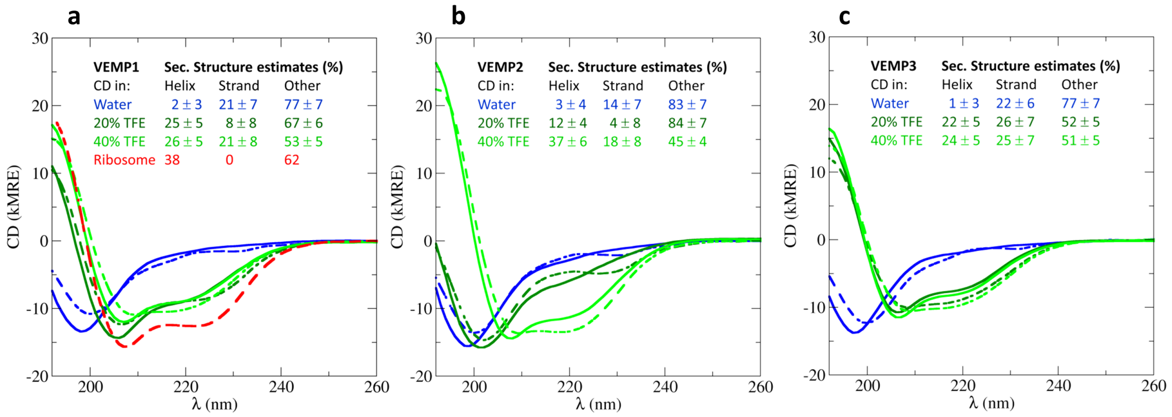

Figure S2: Measured CD spectra (solid lines) and CD spectra predicted from estimated secondary-structure compositions (dashed lines) for VemP-1 (a), VemP-2 (b) and VemP-3 (c). The CD spectrum predicted for VemP-1 in the ribosome (red dashed line) is calculated from the secondary structure of VemP in the cryo-EM structure.

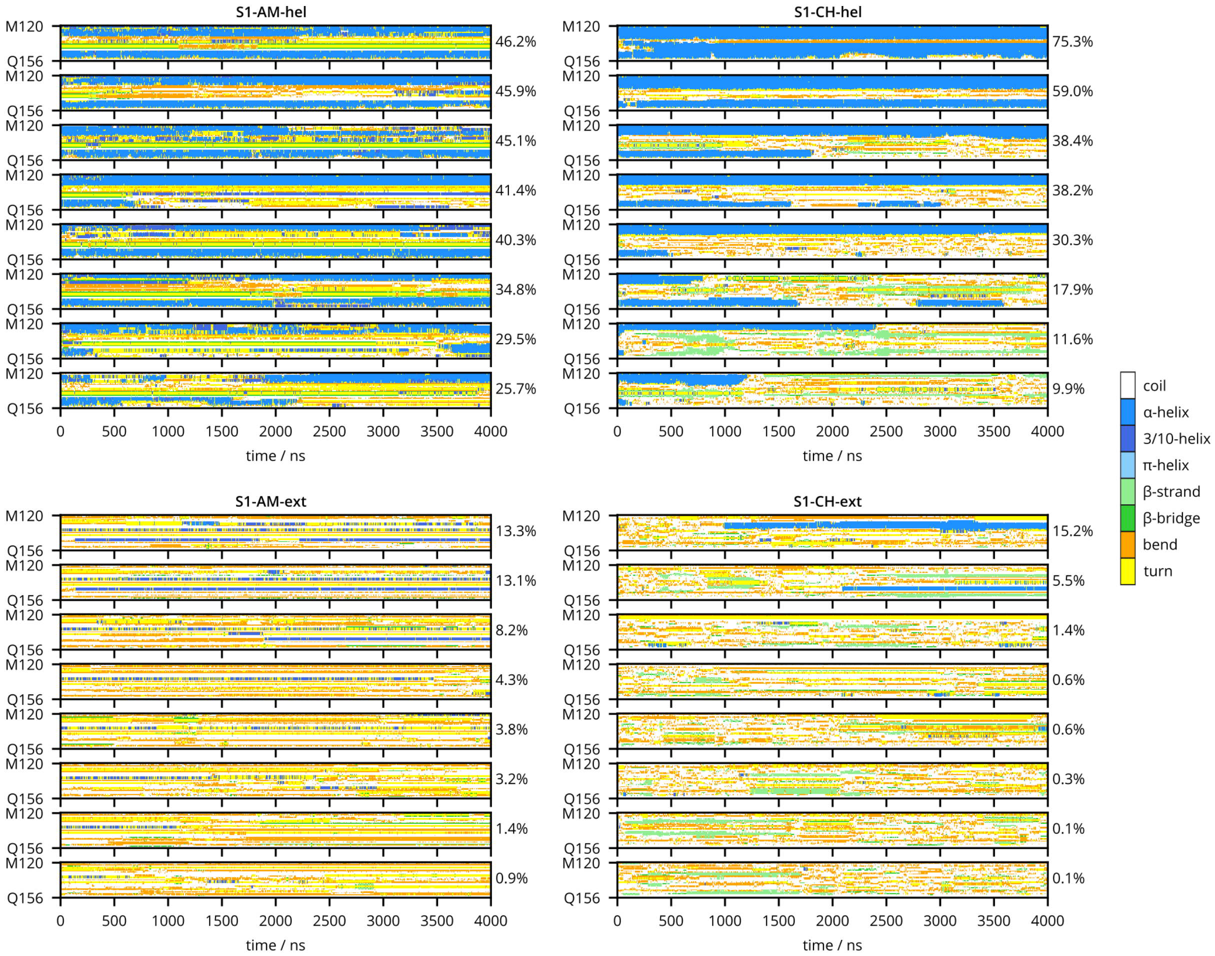

Figure S3: Secondary structure classification of VemP-1 in water solution defined by DSSP algorithm as a function of simulation time. The plot codes refer to the AMBER (AM) or CHARMM (CH) force fields and the start of the simulation from a helical (hel) or extended non-helical (ext) conformations of VemP-1. The timelines are sorted according to the overall helical content, which covers the α-helical and 3/10-helical motives and is shown on the right in %. No TFE was present in the MD simulations.

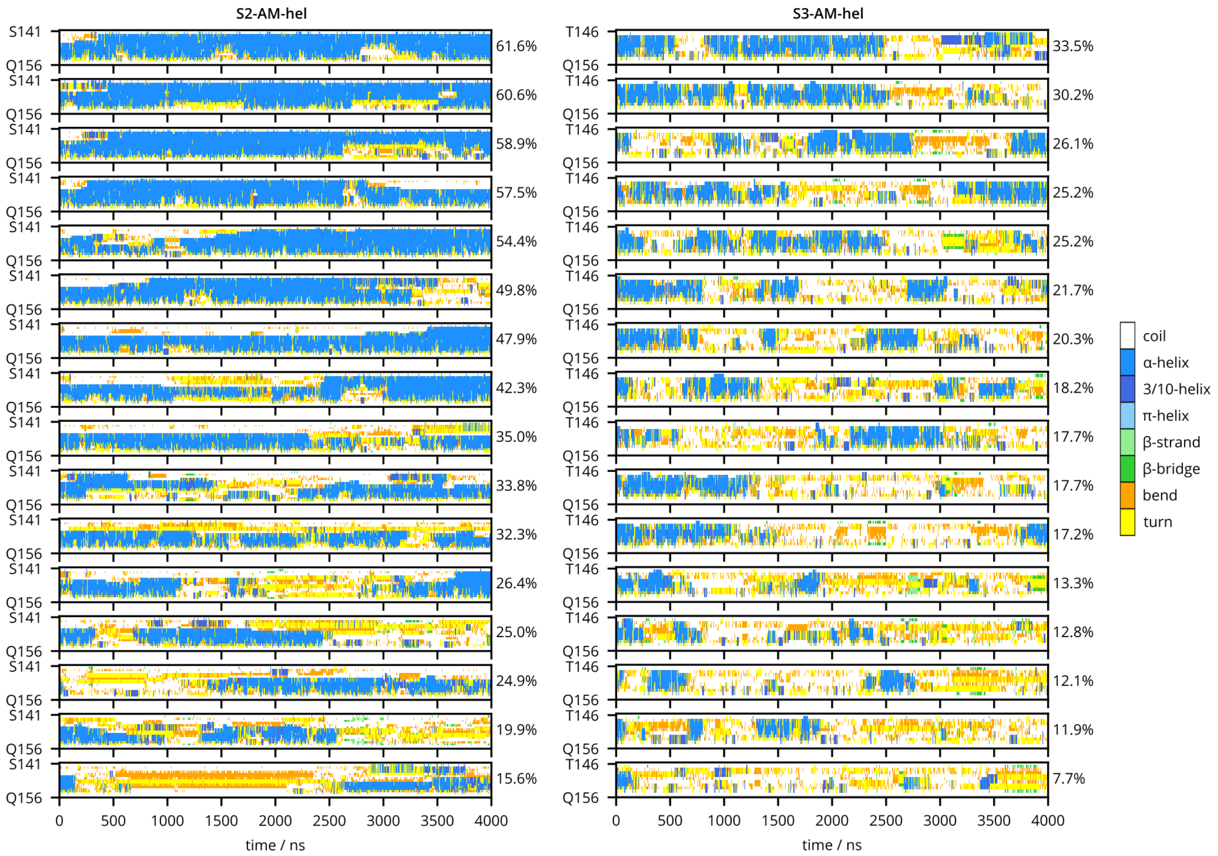

Figure S4: Secondary structure classification of VemP-2 and VemP-3 in solution defined by DSSP algorithm as a function of simulation time. The plot codes refer to the use of AMBER (AM) force field and the start of the simulation from a helical VemP conformations (hel). The timelines are sorted according to the overall helical content, which covers the α-helical and 3/10-helical motives and is shown on the right in %. No TFE was present in the MD simulations.

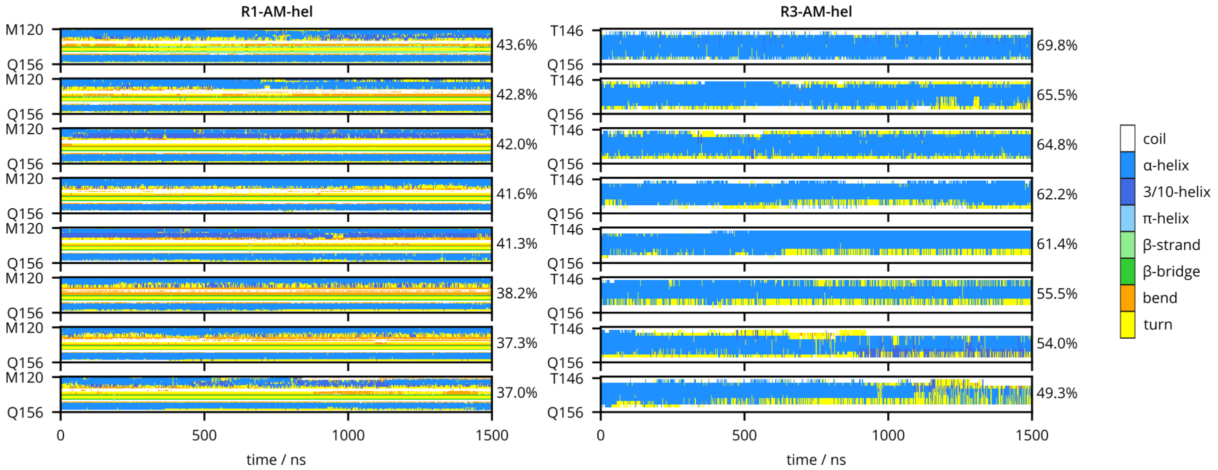

Figure S5: Secondary structure classification of VemP1-1 and VemP-3 in the ribosome tunnel defined by DSSP algorithm as a function of simulation time. The plot codes refer to the use of AMBER (AM) force field and the start of the simulation from a helical VemP conformations (hel). The timelines are sorted according to the overall helical content, which covers the α-helical and 3/10-helical motives and is shown on the right in %. No TFE was present in the MD simulations.

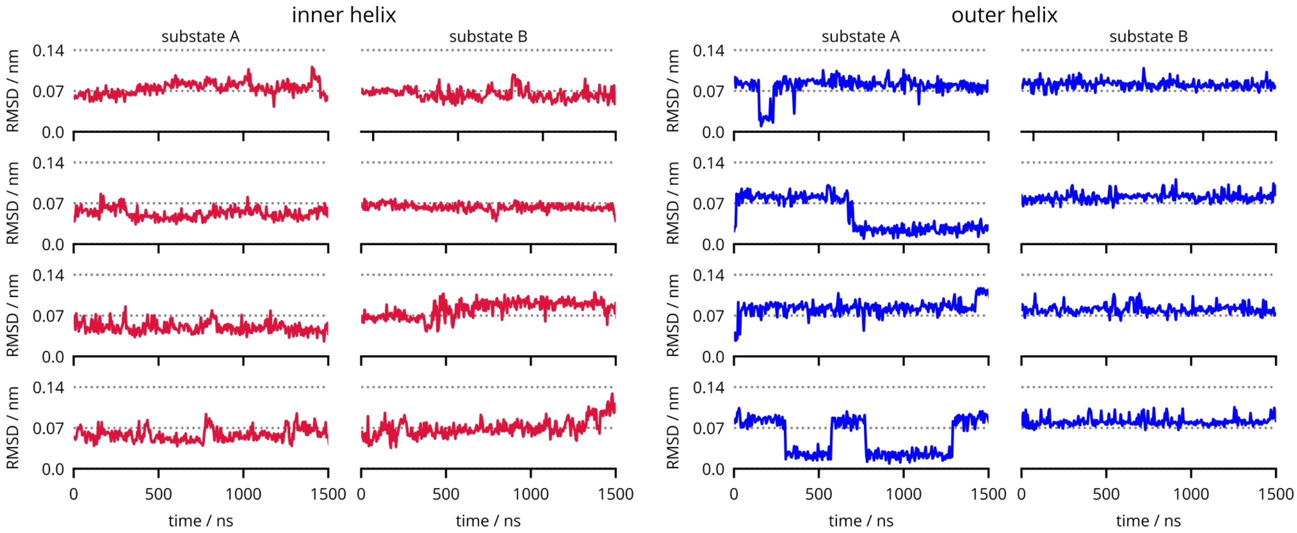

Figure S6: Backbone root-mean-square deviation (RMSD) of the two helices of VemP-1 in the ribosome tunnel after the least-square self-alignment as a function of simulation time.

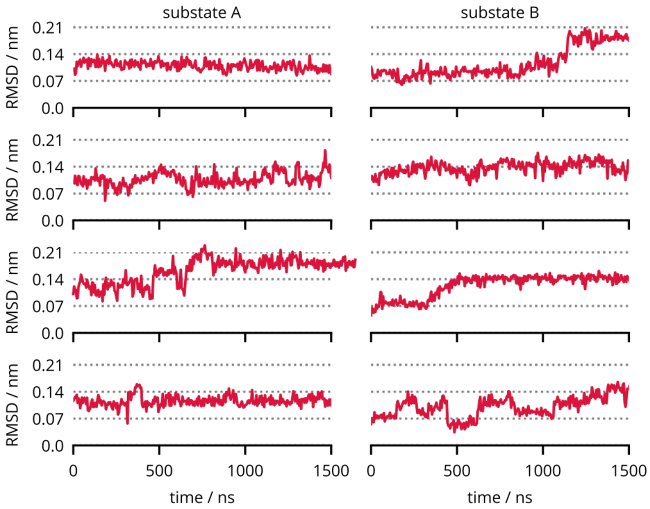

Figure S7: Backbone root-mean-square deviation (RMSD) of VemP-3 (inner helix) in the ribosome tunnel after the least-square self-alignment as a function of simulation time.

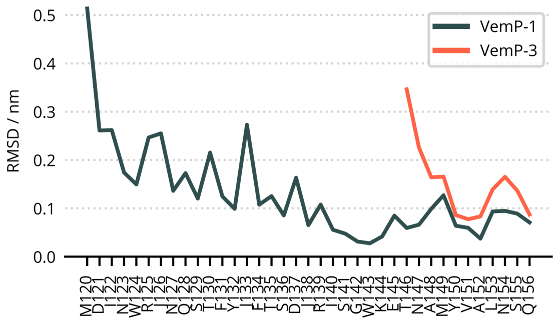

Figure S8: Residue-wise RMSD between cryo-EM and MD models of VemP-1 and VemP-3 in the ribosome tunnel as a function of simulation time. Non-hydrogen atoms were used for RMSD calculation after least-square alignment of the tunnel residues (Tab. S1). The values were calculation for the MD models obtained as averages of all RNC trajectories. No TFE was present in the MD simulations.
